# Experimental quantum fingerprinting with weak coherent pulses

**DOI:** 10.1038/ncomms9735

**Published:** 2015-10-30

**Authors:** Feihu Xu, Juan Miguel Arrazola, Kejin Wei, Wenyuan Wang, Pablo Palacios-Avila, Chen Feng, Shihan Sajeed, Norbert Lütkenhaus, Hoi-Kwong Lo

**Affiliations:** 1 Center for Quantum Information and Quantum Control, University of Toronto, Toronto, Ontario, Canada M5S 3H6; 2 Department of Electrical and Computer Engineering, University of Toronto, Toronto, Ontario, Canada M5S 3G4; 3 Department of Physics, University of Toronto, Toronto, Ontario, Canada M5S 1A7; 4 Institute for Quantum Computing, University of Waterloo, 200 University Avenue West, Waterloo, Ontario, Canada N2L 3G1; 5 Department of Physics and Astronomy, University of Waterloo, 200 University Avenue West, Waterloo, Ontario, Canada N2L 3G1; 6 School of Science and State Key Laboratory of Information Photonics and Optical Communications, Beijing University of Posts and Telecommunications, Beijing 100876, China; 7 Department of Physics, University of Hong Kong, Pokfulam Road, Hong Kong, China; 8 Faculty of Science, National University of Engineering, Lima 15333, Peru; 9 School of Engineering, University of British Columbia, Kelowna, British Columbia, Canada V1V 1V7; 10 Department of Electrical and Computer Engineering, University of Waterloo, 200 University Avenue West, Waterloo, Ontario, Canada N2L 3G1

## Abstract

Quantum communication holds the promise of creating disruptive technologies that will play an essential role in future communication networks. For example, the study of quantum communication complexity has shown that quantum communication allows exponential reductions in the information that must be transmitted to solve distributed computational tasks. Recently, protocols that realize this advantage using optical implementations have been proposed. Here we report a proof-of-concept experimental demonstration of a quantum fingerprinting system that is capable of transmitting less information than the best-known classical protocol. Our implementation is based on a modified version of a commercial quantum key distribution system using off-the-shelf optical components over telecom wavelengths, and is practical for messages as large as 100 Mbits, even in the presence of experimental imperfections. Our results provide a first step in the development of experimental quantum communication complexity.

## Abstract (web summary)

Quantum communication allows exponential reductions in the information that must be transmitted to solve distributed computational tasks. Here, the authors demonstrate with weak coherent optical pulses a quantum fingerprinting system that transmits less information that the best known classical protocol.

## Graphical abstract

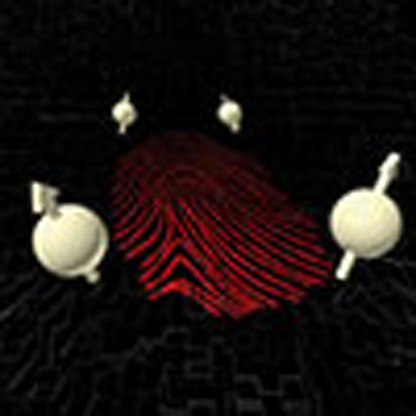

What technological advantages can be achieved by directly harnessing the quantum-mechanical properties of physical systems? In the context of communications, it is known that quantum mechanics enables several remarkable improvements, such as cryptographic protocols that are classically impossible^1^,^2^,^3^, enhanced metrology schemes^4^ and reductions in the communication required between distributed computing devices^5^,^6^,^7^,^8^,^9^,^10^,^11^,^12^,^13^. And yet, despite our advanced understanding of what these quantum advantages are, demonstrating them in a practical setting continues to be an outstanding and central challenge. Important progress has been made in this direction^14^,^15^,^16^,^17^,^18^,^19^,^20^,^21^,^22^, but many cases of quantum improvements have never been realized experimentally.

An important example of a quantum advantage occurs in the field of communication complexity: the study of the minimum amount of information that must be transmitted to solve distributed computational tasks^5^,^6^,^7^,^8^. It has been proven that for several problems, quantum mechanics allows exponential reductions in communication compared with the classical case^7^,^9^,^10^,^11^,^12^,^13^. These results, besides being of great fundamental interest^6^,^7^,^23^, have important practical applications for the design of communication systems, very-large-scale integration circuit design and data structures^24^.

There are two types of communication complexity problems. The first one is to minimize the amount of information that must be transmitted to solve a task, and the second one is to minimize the error probability to solve a task with a fixed amount of transmitted information. These two problems are really two sides of the same coin, since any given protocol requires a certain amount of transmitted information to reach a given error probability. However, conceptually and experimentally, they belong to different regimes. To date, only a few proof-of-principle implementations of quantum communication complexity protocols have been reported^25^,^26^,^27^. For instance, ref. 27 was the first experiment that demonstrated an advantage of quantum over classical communication for the second problem, even without entanglement. However, all such experiments have faced daunting scalability issues, limiting their results to a quantum advantage for the second problem only, with the transmitted information restricted to single qubits. Up until now, a quantum advantage for the first problem, a reduction in the transmitted information compared with the classical case—which is the central issue in quantum communication complexity^7^—has not yet been demonstrated.

Quantum fingerprinting is arguably the most appealing protocol in quantum communication complexity, as it constitutes a natural problem for which quantum mechanics permits an exponential reduction in the transmitted information^9^,^28^,^29^. In this problem, Alice and Bob are each given an *n*-bit string, which we label *x* and *y*, respectively. In the simultaneous message passing model^5^, they must each send a message to a third party, the referee, whose task is to decide whether the inputs *x* and *y* are equal or not with an error probability of at most ε. Alice and Bob do not have access to shared randomness and there is only one-way communication to the referee. It has been proven that any classical protocol for this simultaneous message passing problem must transmit at least 
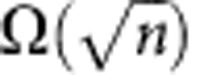
 bits of information to the referee for a desired error probability^30^,^31^. On the other hand, using quantum communication, Alice and Bob only need to transmit *O*(log_2_ *n*) qubits of information to solve the problem with the same error probability. Therefore, for the specific goal of reducing the transmitted information, quantum communication provides an exponential improvement over the classical case^9^.

25,26 have reported heroic attempts at the implementation of quantum fingerprinting. Nonetheless, as noted already in ref. 25, a serious drawback of these approaches is that their fingerprint states must be highly entangled. As a result, even for low input sizes, the experimental requirements greatly exceed that which is possible to achieve with current technology. For this reason, the implementations of refs 25, 26 are restricted to one single-qubit transmission and within a few metres, without a practical possibility of scaling them to demonstrate a reduction in the transmitted information.

In this work, we present a proof-of-concept experimental demonstration of a quantum fingerprinting system over a 5-km standard fibre operating at telecom wavelengths. The protocol is practical for input sizes as large as 100 Mbits. Crucially, our system is capable of transmitting less information than the best-known classical protocol for the fingerprinting problem. Our system is based on the quantum fingerprinting protocol with weak coherent states of ref. 29. Although this protocol is already practical, we overcome various challenges to its experimental implementation. First, we develop an efficient error-correction algorithm that allows us to substantially relax the requirements on the experimental devices and reduce the running time of the protocol. Second, we use an improved decision rule for the referee compared with the one used in ref. 29. Finally, we perform detailed simulations of the protocol that allows us to identify the appropriate parameters for performing the experiment. This enables us to run the protocol using commercial off-the-shelf components. Indeed, we implemented the protocol by using a commercial plug and play system originally designed for quantum key distribution (QKD)^32^, to which we added several important modifications. We also characterized the system and showed that, within our theoretical model of the experiment, its performance is consistent with achieving the desired error probability. Finally, we experimentally tested the system for input sizes of up to 100 Mbits and obtained data that are consistent with the protocol transmitting less information than the best-known classical protocol.

## Results

### Coherent-state quantum fingerprinting protocol

**Figure 1:**
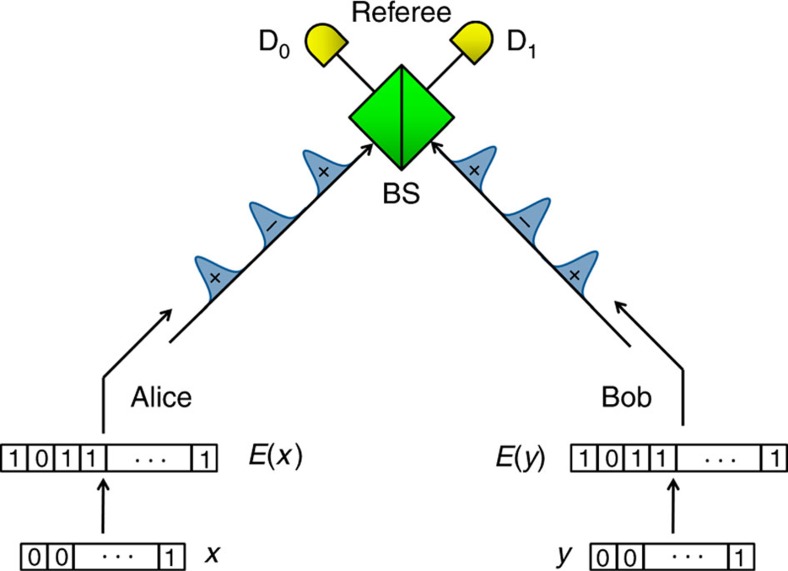
A schematic illustration of the quantum fingerprinting protocol. Alice and Bob receive inputs *x* and *y*, respectively, which they feed to an ECC to produce the codewords *E*(*x*) and *E*(*y*). Using these codewords, they modulate the phases of a sequence of coherent pulses that they send to the referee. The incoming signals interfere at a beam splitter (BS) and photons are detected in the output using single-photon detectors *D*_0_ and *D*_1_. In an ideal implementation, detector *D*_1_ fires only when the inputs to Alice and Bob are different.

In the quantum fingerprinting protocol of ref. 29, portrayed in Fig. 1, Alice first applies an error-correcting code (ECC) *E*:{0,1}^*n*^→{0,1}^*m*^ to her input *x* of *n* bits. This results in a codeword *E*(*x*) of 
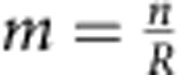
 bits, which she uses to prepare a sequence of *m* coherent states, where 
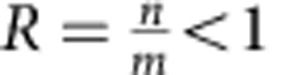
 is the rate of the code. This sequence of coherent states is given by the state


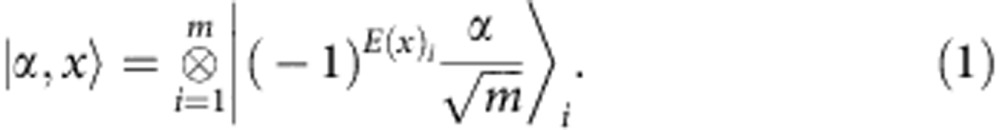


Here *E*(*x*)_*i*_ is the *i*th bit of the codeword and *α* is a complex amplitude. Notice that all the coherent states have the same amplitude, but their individual phases depend on the particular codeword, which in turn is determined by the input *x*. The total mean photon number in the entire sequence is *μ*:=|*α*|^2^, which in general depends on the length of the codewords *m*.

In our protocol, the encoded fingerprinting states are coherent states, instead of single-photon states as required in previous schemes^25^. Hence, a perfect two-photon interference is not required^33^. All we need is a measurement by the referee that allows her to verify whether the relative phases of the incoming pulses are equal or different. A way of achieving this consists of a phase interferometer in which the individual pulses enter a balanced beam splitter, and whenever there is a click in the output detectors, it is unambiguously revealed whether their phases are the same or not^34^.

Indeed, in our scheme, Bob does the same as Alice for his input *y*, and they both send their sequence of states to the referee, who interferes the individual states in a balanced beam splitter. The referee checks for clicks at the outputs of the phase interferometer using single-photon detectors, which we label ‘*D*_0_' and ‘*D*_1_'. In the ideal case, a click in detector *D*_1_ will never happen if the phases of the incoming states are equal, that is, if *E*(*x*)_*i*_⊕*E*(*y*)_*i*_=0. However, it is possible for a click in detector *D*_1_ to occur if the phases are different, that is, if *E*(*x*)_*i*_⊕*E*(*y*)_*i*_=1. Thus, if *x*≠*y*, we expect a number of clicks in *D*_1_ that is proportional to the total mean number of photons and the Hamming distance between the codewords. This allows the referee to distinguish between equal and different inputs by simply checking for clicks in detector *D*_1_.

In ref. 29, it was proven that the quantum information *Q* that can be transmitted by sending the states of equation (1) satisfies


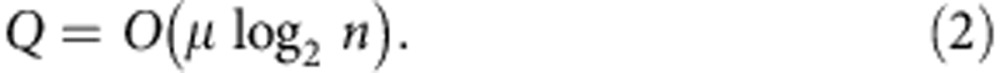


For fixed *μ*, this corresponds to an exponential improvement over the classical case, where 
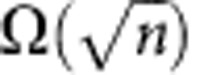
 bits of information must be transmitted^30^,^31^. It is precisely in terms of this reduction in the transmitted information that the quantum protocol provides an advantage over the classical case.

The states of equation (1) can be thought of as a coherent-state version of the encoding of an *m*-dimensional state into the state of a single photon across *m* modes, as discussed in depth in ref. 35. Essentially, by fixing the total mean photon number to a constant, we are restricting ourselves to an exponentially small subspace of the larger Hilbert space associated with the optical modes, which in turn restricts the capability of these systems to transmit information. Thus, to achieve the central goal of a reduction in the transmitted information, our protocol must use a number of modes that is linear in the input size *n*, with the benefit that the total mean photon number *μ* is independent of input size and therefore very small.

Finally, we remark that a quantum protocol without entanglement or two-photon interference was demonstrated previously in ref. 27. The demonstration in ref. 27 utilized polarization qubits to tackle the communication complexity problem of maximizing the probability of solving the modulo-4 sum problem^8^ with a restricted amount of transmitted information. In principle, both ref. 27 and our current paper can use coherent pulses and a phase interferometer. However, from a physical point of view, since the aims of our work and ref. 27 were different, the underlying physics also has some differences. Our protocol employed states of large dimension to encode more classical information, while ref. 27 used coherence properties of qubits, which were fixed to a two-dimensional system without interactions among the states. To use large dimensionality, we utilize time bins with phase encoding and perform an interaction of the states with a phase interferometer.

### Protocol in the presence of experimental imperfections

In the presence of experimental imperfections such as detector dark counts and optical misalignment, detector *D*_1_ may fire even when the inputs are equal. Therefore, it does not suffice to check for clicks in this detector—we must introduce a different decision rule for the referee. The decision rule proposed in ref. 29, which is based on the fraction of clicks that occur in detector *D*_1_, is extremely sensitive to experimental imperfections. Instead, in this work we construct a better decision threshold based only on the total number of clicks observed in detector *D*_1_.

Let *D*_1,E_ and *D*_1,D_ be random variables corresponding to the number of clicks in detector *D*_1_ for the case of equal and worst-case different inputs, respectively. It can be shown that these distributions can be well approximated by binomial distributions *D*_1,E_∼*Bin*(*m*,*p*_E_) and *D*_1,D_∼*Bin*(*m*,*p*_D_), where *m* is the number of modes and *p*_E_, *p*_D_ are the probabilities of observing a click in each mode for the case of equal and worst-case inputs, respectively. These probabilities are given by ref. 29:


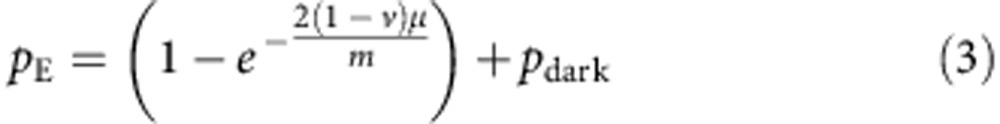



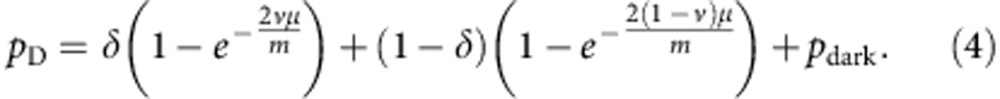


Here *ν* is the interference visibility—which quantifies the contrast of the interferometer—and *p*_dark_, the dark count probability, is the probability that a detector will fire even when no incident photons from the signals are present. As before, *μ* is the total mean photon number in the signals and *δ* is the minimum distance of the ECC, which is defined as the smallest relative Hamming distance between any two distinct codewords.

**Figure 2:**
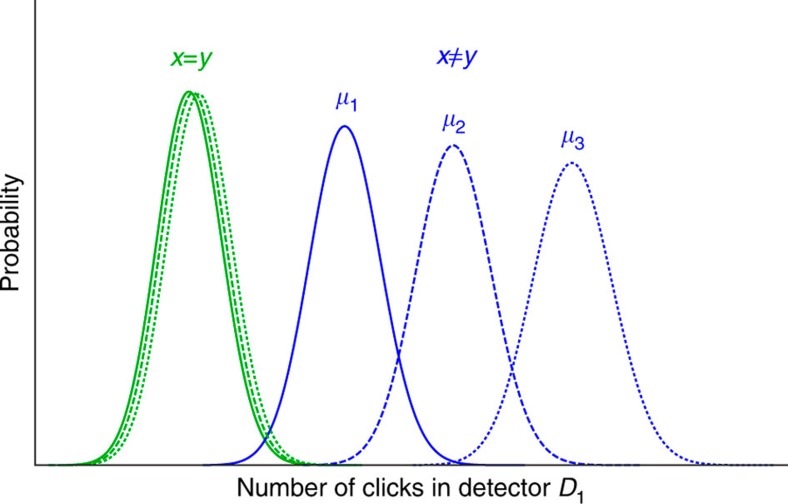
An illustration of the probability distributions for the total number of clicks observed in detector *D*_1_ for equal inputs and worst-case different inputs. The distributions are shown for three different total mean photons numbers: *μ*_1_ (solid), *μ*_2_ (dashed) and *μ*_3_ (dotted), where *μ*_1_<*μ*_2_<*μ*_3_. For illustration, values of *μ*_1_=617, *μ*_2_=693 and *μ*_3_=776 were chosen for the figure. The distributions for equal inputs (green) are dominated by dark counts, so they are largely unaffected by the changes in *μ*. On the other hand, for the worst-case different inputs (blue), the mean value of the distributions depends strongly on *μ*. Therefore, the error in distinguishing both distributions can be controlled by choosing *μ* appropriately.

The referee sets a threshold value *D*_1,th_ such that, if the number of clicks is smaller than or equal to *D*_1,th_, he will conclude that the inputs are equal. Otherwise, he concludes that they are different. Note that, unlike the ideal case, in the presence of imperfections, an error can occur even when the inputs are equal. In our protocol, the value of *D*_1,th_ is chosen in such a way that an error is equally likely to occur in both cases, so that the probability of error is given by


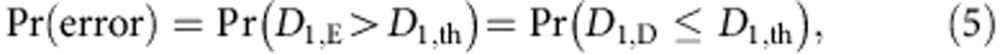


which can be calculated directly from the distributions of *D*_1,E_ and *D*_1,D_. This is illustrated in Fig. 2. In general, for each input size *n*, the total mean photon number *μ* is uniquely determined by finding the value of *μ* such that Pr(error)≤*ε*, where *ε* is the desired error probability of the protocol.

Note that this model is expected to be correct as long as the parameters quantifying the experimental imperfections as well as the mean photon number *μ* are all constant during the run of the protocol. In practice this is not necessarily the case, so our model should be understood as an approximation of the actual performance of the system.

Finally, we note that in any implementation of the protocol there will be some loss captured by the combined effect of limited detector efficiency and channel loss. We quantify this with the single parameter *η*<1. As shown in ref. 29, the effect of loss can be compensated by adjusting the total mean photon number accordingly: *μ*→*μ*/*η*. Thus, the protocol is robust to loss.

### Error-correcting code

In quantum fingerprinting, an ECC is used to amplify the Hamming distance between the inputs of Alice and Bob. Even if these inputs are originally very close to each other—for example, if they differ in a single position—after applying the ECC, the resulting codewords will have a much larger Hamming distance. In the worst-case scenario, this distance is given by the minimum distance of the code. Note that an important difference between a standard classical error-correction implementation and our current implementation is that in our implementation, Alice and Bob only need to perform encoding, but not the decoding of the ECCs. For this reason, we are concerned only with the computational complexity in encoding. This greatly simplifies our requirements.

**Figure 3:**
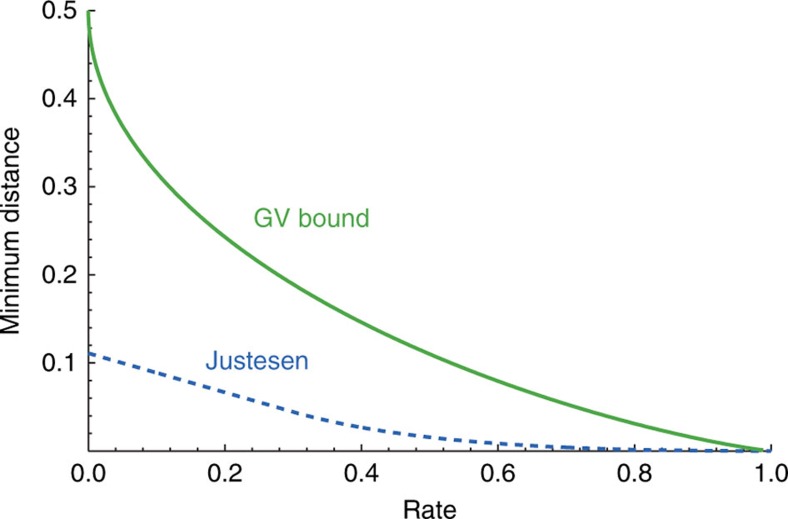
The Gilbert–Varshamov bound compared with the distance–rate relationship achieved by Justesen codes. For various rates, a code satisfying the Gilbert–Varshamov bound—like the one achieved in this paper—provides a minimum distance that is more than three times the value for Justesen codes, which were used in previous works^9^,^29^.

The quantum fingerprinting protocol of ref. 29 used Justesen codes as an example to illustrate the properties of the protocol. However, these codes are not optimal for quantum fingerprinting. Here we construct a more efficient ECC that significantly relaxes the requirements on the experimental devices and leads to a faster implementation of the protocol. We make use of a subclass of random linear codes (RLCs)^36^ whose generator matrices are Toeplitz matrices. Our ECC can asymptotically approach the Gilbert–Varshamov bound^37^,^38^. For various rates, it provides a minimum distance that is more than three times the value for Justesen codes. This is clearly illustrated in Fig. 3. The implementation details of our ECC are shown in Methods.

### Experimental set-up

We demonstrate our proof-of-concept quantum fingerprinting protocol using a plug and play scheme^39^, initially designed for QKD. The advantage of the plug and play system with respect to other viable systems is that it offers a particularly robust and stable implementation. This allows us to perform reliable experiments with highly attenuated coherent states for long time durations. We implement the protocol on top of two commercial systems, namely ID-500 and Clavis2, manufactured by ID Quantique.

**Figure 4:**
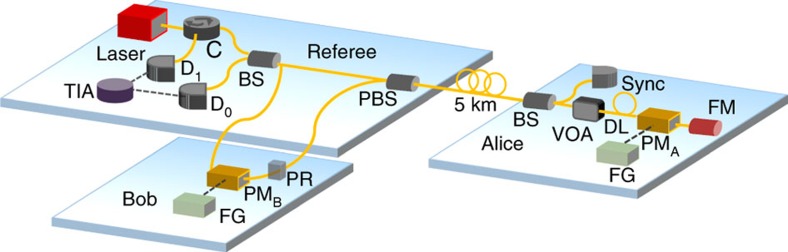
Experimental set-up for quantum fingerprinting. The laser source at the referee's set-up emits photon pulses at about 1,551 nm, which are separated at a 50:50 beam splitter (BS) into two pulses, the signal pulse and the reference pulse. The signal pulse passes through Bob's phase modulator (PM) and then through a polarization rotator (PR), which rotates the pulses' polarization by 90°. The pulses are then recombined at a polarization beam splitter (PBS) where they exit through the same port and travel to Alice through the 5-km fibre. After passing through Alice's BS, the reference (forward) pulse is split into two pulses, where one is used as a synchronization (Sync) and the other one continues travelling. Similarly, the signal (backward) pulse is split into two. Then, Alice uses her PM to set the phase of the signal pulse only, according to her codeword *E*(*x*). Once the reference and the signal pulses are reflected back by the Faraday mirror (FM), she attenuates them to the desired photon level by using the VOA. When the two pulses return in the direction of the referee, because of Alice's FM, the reference pulse will travel through Bob, who uses his PM to modulate the pulse according to his codeword *E*(*y*). Both Alice and Bob use two external function generators (FG) to control the PMs. Finally, the two pulses arrive simultaneously at the BS, where they interfere and are detected by two detectors *D*_0_ and *D*_1_. The detection events are recorded by a time interval analyser (TIA).

In our set-up, which is shown in Fig. 4, the referee starts by sending two strong pulses at about 1,551 nm to Alice over a 5-km fibre. Once the two pulses reach Alice, she uses the reference pulse as a synchronization signal to activate her phase modulator, which she employs to set the phase of the signal pulse according to her codeword *E*(*x*). Both pulses are reflected back by a Faraday mirror, which rotates the pulses' polarization by 90, and she attenuates them to the desired photon level using the variable optical attenuator (VOA). Once the pulses return back, due to the Faraday mirror, the pulses take opposite paths, such that the reference pulse now passes through Bob and its phase is modulated by Bob's phase modulator according to *E*(*y*). Finally, the two pulses interfere at the referee's beam splitter and the detection events are registered using two high-quality single-photon detectors *D*_0_ and *D*_1_. It is important to note that the returning signal pulse modulated by Alice travels directly to the referee, while the returning reference pulse passing through Bob does not contain any information about Alice's codeword. This guarantees that there is no communication between Alice and Bob.

Since the operating conditions of our protocol are significantly different from those of standard QKD, using a commercial QKD equipment for our implementation requires several important modifications to the system. First, two single-photon detectors—ID220 (manufactured by ID Quantique)—with low dark count rates were installed. Second, we performed several calibration and synchronization processes to enable the system work at an ultra-low mean photon number level, which is about four orders of magnitude lower than those typically used for QKD. Finally, we implemented two external function generators (Agilent 88250A) loaded with the codewords to control Alice's and Bob's phase modulator. The details of our modifications are presented in Methods. We observed high interference visibility of about (99±0.5)% after careful calibration.

### Experimental results

We perform the proof-of-concept quantum fingerprinting experiment over a standard telecom fibre of 5 km between Alice and the referee. The overall loss between the output of Alice's VOA and the input of the referee's detector *D*_1_—which includes the losses of quantum channel, polarization beam splitter, beam splitter and the circulator—is about 3 dB (2.36 dB) for ID-500 (Clavis2). The channel between Bob and the referee is about a few metres, and its overall loss including Bob's channel, the beam splitter and the circulator is about 1.5 dB (1 dB). We summarize all system parameters in Table 1. On the basis of these parameters, for a given input size *n*, we use our model of the protocol to optimize the photon number *μ* to achieve a desired error probability *ε*.

Because there is loss in the channels and the detectors are not perfectly efficient, Alice and Bob must use higher mean photon numbers compared with the case with no channel loss and with perfect detectors. As implied by equation (2), this also leads to an increase in the transmitted information, which we take into account in our calculations of the transmitted information. In particular, if Alice and Bob experience different amounts of loss, they must choose a different mean photon number when preparing their signals, ensuring that the amplitude of their pulses is equal when they interfere in the referee's beam splitter.

In the experiment, the detection events registered on *D*_0_ and *D*_1_ in conjunction with the known experimental conditions in the system can be used to characterize the photon numbers sent out by Alice and Bob, the dark count probability and the visibility of the interferometer. From the characterization of these parameters, we find that there is a good agreement with our model of the system. The main source of uncertainty is due to an imperfect matching between the observed mean photon numbers and those pre-calibrated from the VOA. This uncertainty is determined by the fluctuations of several devices, such as laser power, VOA and detector efficiency. The detailed values of this uncertainty are shown in Methods.

The quantum fingerprinting protocol is tested over several values of the input size *n*. For each *n*, we record the detection counts on *D*_1_ for two types of input data: equal inputs *E*(*x*)=*E*(*y*), and the worst-case different inputs, that is, those for which the codewords *E*(*x*)≠*E*(*y*) have a distance equal to the minimum distance. For our experiment, we minimize the transmitted information by choosing an optimal value of *δ*=0.22 for the minimum distance. From the threshold value *D*_1,th_ that is pre-calculated from our model, the referee can distinguish between equal and different inputs. The upper bound *Q* on the quantum information Alice and Bob is calculated from their respective mean photon numbers *μ*_A_ and *μ*_B_, as well as the codeword length *m*.

**Figure 5:**
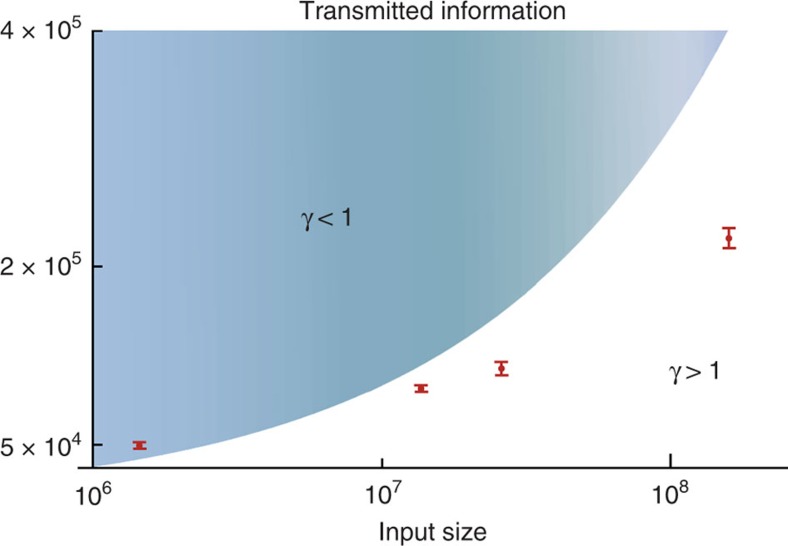
Log-linear plot of the total transmitted information by Alice and Bob in our protocol. The blue area indicates the region where the classical protocol transmits less information than our protocol, while the red points show our experimental results. The quantum advantage *γ* corresponds to the ratio between the transmitted classical information and the upper bound on the transmitted quantum information. The error bars correspond to one standard deviation. For large *n*, our results are strictly better than the best-known classical protocol for a range of practical values of the input size.

In Fig. 5, we show the transmitted information as a function of the input size *n* for an error probability of *ε*=5 × 10^−5^. An error of 5 × 10^−5^ was chosen because it was the lowest error probability that was achieved by all runs of the experiment. The error probability was calculated from our theoretical model of the experiment. Within experimental uncertainty, the worst-case values of the mean photon number, visibility and dark count probability were used to reconstruct the probability distributions of clicks in detector *D*_1_. These distributions, in turn, were used to calculate the error probability from equation (5). Since our theoretical model is only an approximation, the error probability should also be understood as approximate. The blue area in Fig. 5 indicates the region where the best-known classical protocol of ref. 30 transmits less information than our quantum protocol. For this target error probability, the classical protocol requires the transmission of 
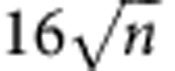
 bits. The red points show our experimental results, where the data point for the largest *n* is obtained from ID-500 and the other three data points are obtained from Clavis2. Note that Clavis2 and ID-500 have almost the same optics and functionality. We use the same measurement and processing method for the data obtained from these two systems, and show the experimental results together in one figure instead of two. The error bars come from the uncertainty in the estimation of the mean photon number *μ*. For large *n*, our experimental results are strictly better than those of the classical protocol for a wide range of practical values of the input size.

To obtain further insight into our results, we define the quantum advantage *γ* as the ratio between the transmitted classical information *C* of the best-known classical protocol^30^ and the upper bound *Q* on the transmitted quantum information:


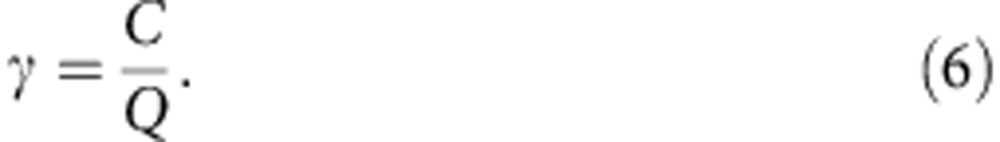


**Figure 6:**
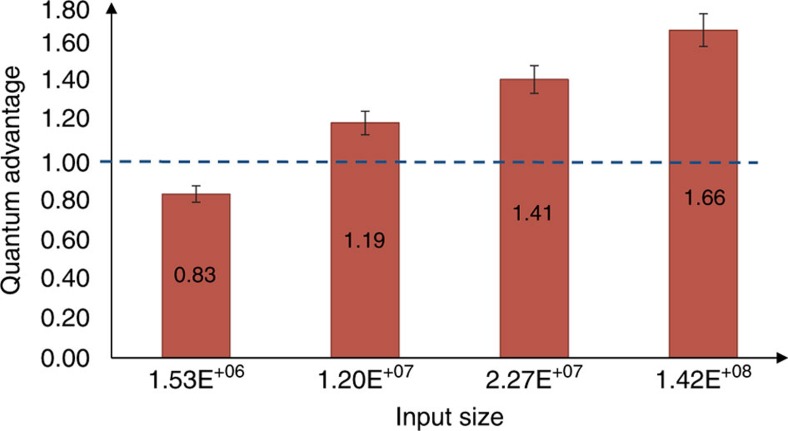
Quantum advantage *γ* between the transmitted classical information and the upper bound on the transmitted quantum information. The uncertainty refers to one standard deviation, which mainly comes from the error in estimating the mean photon number per pulse. For the three large input sizes, the ratio is well above 1. The quantum advantage was as large as *γ*=1.66, which implies that the transmitted information in the classical protocol was 66% larger than in the quantum case.

A value *γ*>1 for a given error probability *ε* implies that less information is transmitted in the quantum case than in the classical one. This allows us to use the quantum advantage as a figure of merit to assess the performance of our quantum fingerprinting implementation. In Fig. 6, we show the experimental results for *γ* as a function of different input sizes. For the three largest input sizes, the ratio is well above 1, and the classical protocol transmitted as much as 66% more information than the quantum protocol. For the smallest input size, no quantum improvement was obtained.

## Discussion

On the basis of the protocol of ref. 29, we have experimentally demonstrated a proof-of-concept quantum fingerprinting system that is capable of transmitting less information than the best-known classical protocol for this problem. Our experimental test of this system indicates that its operation is consistent with our model of the devices and hence also with achieving the desired error probability. Moreover, we have operated our system in a parameter regime in which the information transmitted in the protocol is up to 66% lower than the best-known classical protocol. This constitutes the first time that a quantum fingerprinting protocol has been carried out that is capable of achieving this reduction in the transmitted information.

It is an appealing and useful property of this quantum fingerprinting protocol that we can achieve a quantum advantage without the need for entanglement, single-photon sources or squeezing. Where does the improvement come from? As discussed extensively in ref. 35, the states of equation (1) that are used in our protocol are a coherent-state version of an encoding of *m*-dimensional quantum states into states of a single photon across *m* modes. Through this encoding, exponentially more ‘sufficiently distinguishable' quantum states can be fitted into an *O*(log_2_ *m*)-qubit Hilbert space as opposed to orthogonal classical states. In our protocol, instead of *O*(log_2_ *m*) qubits, the same amount of quantum information can be encoded into a sequence of coherent states.

One can understand the quantum advantage as arising from the non-orthogonality of weak coherent states and the quantum-mechanical properties of single-photon detectors. In the protocol, the weak coherent states have a very low mean photon number. This means that the two possible states that are sent in each mode, 
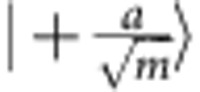
 and 
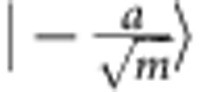
, are highly non-orthogonal and fundamentally difficult to distinguish. Therefore, very little information can be learnt by looking at each pulse. This is essentially the reason why the transmitted information is very low—exponentially less than in the classical case. On the other hand, after the coherent states interfere in the beam splitter, a click in the single-photon detector unambiguously provides valuable information to the referee: she now knows whether the phases of the coherent states are equal or not. This unambiguous information is only possible because the detectors respond quantum mechanically to the incoming light field.

The main goal of our experiment is to demonstrate a reduction in the transmitted information compared with the best-known classical protocol. However, from a practical perspective, one might be interested in additional quantities, such as energy expenditures or running time, beyond the abstract transmitted information. In our protocol, the running time is quadratically larger than in the classical case, provided we ignore the running time required for the ECC, which is the dominant one. Therefore, if running time during communication is a priority, our protocol has a disadvantage: the quantum protocol may become infeasible for a very large input size of time bins, limited by the repetition rate of the laser source. Nonetheless, if minimizing energy expenditures is a priority, our protocol offers a significant advantage. In particular, the number of photons used is more than quadratically smaller than in a classical protocol using photonic bits, where 
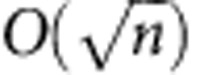
 photons are needed compared with *O*(1) photons in the quantum case.

Finally, in this work, we have tested our model of the system and used that test to make an indirect assessment of the error probability based on our theoretical model. Future implementations should improve on this by treating the system as a black box, using the data directly to make statistical inferences about the error probability, without relying on an approximate model of the system. Overall, it is remarkable that quantum fingerprinting can be realized while revealing only a very small amount of information to the referee—a feature of the protocol that may have important applications to fields such as cryptography^40^ and information complexity^41^, where this extremely small leakage of information plays a fundamental role. Our results constitute a significant first step in the development of experimental quantum communication complexity, which may also be extended to other protocols with a proven exponential advantage over the classical case^10^,^11^,^35^,^42^.

## Methods

### Error-correcting code

In quantum fingerprinting protocol, an ECC with a high rate and a large minimum distance is desired, since a higher rate leads to lower transmitted information and larger tolerance for dark counts, while a larger minimum distance leads to smaller error probability for fixed mean photon number. Fundamentally, there is an inherent trade-off between the rate and distance of ECCs. In particular, the Gilbert–Varshamov bound states that there exists some binary linear code whose rate *R* and minimum distance *δ* satisfy the relation


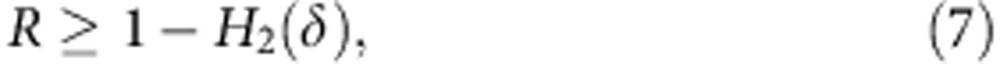


where *H*_2_(·) is the binary entropy function. Using a binary linear code that approaches this bound would constitute a significant improvement over the codes used in previous protocols.

It is well known in coding theory that RLCs can asymptotically approach the Gilbert–Varshamov bound with encoding complexity *O*(*n*^2^) (ref. 43). However, in quantum fingerprinting, the input size *n* is typically very large (for example, *n*=10^8^), thus making the encoding time prohibitively high. To reduce this encoding complexity, we make use of a subclass of RLCs whose generator matrices are Toeplitz matrices. A Toeplitz matrix is a matrix in which each descending diagonal from left to right is constant. An *n* × *m* Toeplitz matrix is completely determined by the *n*+*m*−1 elements on its first row and column. This structure implies that only *O*(*n* log *n*) time for encoding is required for this subclass of RLCs^36^. In addition, these codes also asymptotically approach the Gilbert–Varshamov bound. By using this family of codes, we are able to reduce the encoding times by several orders of magnitude, making them suitable for practical applications.

The exponential separation between quantum and classical communication complexity for the equality function only holds if Alice and Bob do not have access to shared randomness that is generated in each run of the protocol^30^. However, even though the generator matrices of our RLCs are randomly constructed, once they have been created they remain fixed for all future instances of the protocol. This ensures that no new randomness is generated in each run of the protocol, as required to satisfy the conditions of the exponential separation. In particular, Alice and Bob can store the generator matrices in memory and use them to encode their inputs in exactly the same way as if they had been generated deterministically.

For our experiment, an encoder programme written in C++ was built and tested, demonstrating the feasibility of this subclass of RLCs. The free Fast-Fourier Transform library FFTW was used to accelerate multiplications with Toeplitz matrices^44^ and the random numbers to construct the matrices were generated from a quantum random number generator^45^. The results from an optimized encoder are shown in Table 2. As we can see, our encoder is highly practical, can be run on any common lab personal computer (PC) and finishes the encoding in an acceptable time frame for input sizes as large as *n*=3 × 10^8^. Faster encoding times could be obtained by using dedicated hardware.

### Experimental details

We performed several modifications on top of the plug and play system, to implement the quantum fingerprinting protocol. First, two single-photon detectors with low dark count rates were installed. Indeed, as can be deduced from equations (3) and (4), lower dark count rates permit the operation of the system at lower mean photon numbers, which lead to a reduction in the transmitted information. Fortunately, our error-correction codes improve the tolerance of the protocol to dark counts, which permits us to use commercial detectors. We employ two commercial free-running InGaAs avalanche photodiodes—ID220. The dark count rate per 1 ns detection gate is about (3.5±0.2) × 10^−6^ and the corresponding quantum efficiency is about 20%. The detections are recorded by a high-precision time interval analyser (PicoQuant HydraHarp 400). The system was run at a repetition rate of 5 MHz with the detector dead time set at 10 μs. This means that after a click occurred, the following 50 pulses are blocked before the detector is active again. This is not a problem in our experiment because the mean photon number in each pulse is extremely low, therefore, the expected number of undetected photons as a result of this effect is negligible compared with other sources of error.

In addition, new functionalities and control signals were added to the system. On one hand, we use the VOA inside Alice to reduce the mean photon number per pulse down to suitable numbers. These values—in the order of 10^−5^ per pulse—were in fact four orders of magnitude lower than those typically used for QKD. Hence, several calibration processes of the system are required, which imposes particular care in the synchronization of the phase modulation and attenuation signals. On the other hand, commercial QKD systems like Clavis2 have an internal random number generator to set the phase modulations, which does not allow us to modulate the phases according to the pre-generated codewords. We solve this difficulty by using two external function generators (Agilent 88250A) loaded with the codewords to control Alice's and Bob's phase modulator. This requires precise synchronization and calibration procedures to guarantee correct phase modulations.

In the proof-of-concept implementation on ID-500, the random numbers controlling the phase modulations are accessible to users. We use our codewords to replace those random numbers directly. However, after testing for an input data size of *n*=1.42 × 10^8^ on ID-500, an unexpected hardware problem made ID-500 unavailable for further experiments. To further test the feasibility of our protocol for different input sizes, we switched to Clavis2 for measurements. In the implementation on Clavis2, since each function generator has a small memory, for simplicity we load a frame of about 430 random numbers to each function generator and reuse these random numbers. This allows us to create binary sequences with the desired distance *δ* that can be used to test the performance of the system. All the above modifications led to the development of a practical system that is capable of performing quantum fingerprinting.

### Practical considerations

In communication complexity, it is assumed that the parties have unlimited computational power. However, from a practical perspective, it may not always be possible to ignore these computational requirements. In fact, even though the running time during communication of our experiment scales linearly with the input size, the total running time of the protocol is dominated by the time required to run the ECC—which is a crucial component of the protocol. For instance, at a repetition rate of 5 MHz, it takes 5 min to run the communication for an output size of *m*=1.5 × 10^9^. On the other hand, even with the use of RLCs with quasi-linear encoding complexity, more than 1 h is needed to run the encoding algorithm, as seen in Table 2. Therefore, the practical advantages of quantum fingerprinting, in terms of reductions in resource expenditures, will likely be found in a reduction of the number of photons used. This is a major property that our protocol possesses. Indeed, for the largest input size that we tested, *n*=1.42 × 10^8^, a total mean photon number of only *μ*≈7 × 10^3^ was used. Moreover, because the protocol does not require time resolution in the detectors—the referee only cares about the number of clicks, not when they happen—in principle it is possible to run this protocol at very fast rates, limited only by the source repetition rate.

In our quantum fingerprinting protocol, the maximum reduction in the transmitted information depends crucially on the dark count probability and the overall loss in the system. Thus, our results can be directly improved by using detectors with higher efficiency and lower dark counts. This can lead to a quantum fingerprinting protocol that, with the use of available technology^46^, transmits several orders of magnitudes less information than the best-known classical protocol for large input sizes. Even though there is no proof that the best-known classical protocol is optimal, a lower bound for the classical transmitted information was proven in ref. 30. This lower bound states that, for any classical protocol with error probability smaller than 0.01, Alice and Bob must send at least 
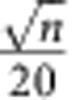
 bits of information. This is roughly two orders of magnitude smaller than the transmitted information of the best-known classical protocol. By using state-of-the-art detectors, it should be possible to demonstrate a quantum fingerprinting protocol capable of beating this classical lower bound. Achieving this would constitute a significant milestone for experimental quantum communication complexity.

Finally, in our implementation, a reference pulse is transmitted between the two participants for a share of synchronization and phase reference. In practice, one can overcome this by using a system where each of Alice and Bob holds a frequency-locked laser source separately. A common phase reference can be established before the start of the protocol or the referee can employ phase-locking techniques to interfere the two pulses from Alice and Bob. Indeed, a potential method for such an implementation is to use the techniques that have been recently developed in the field of QKD^47^,^48^,^49^. This configuration, unlike the plug and play scheme, can also permit Bob to be situated at a large distance from the referee.

### Error probability analysis

We prove that the Toeplitz matrix based RLCs also asymptotically approach the Gilbert–Varshamov bound. Let *G* be a random *n* × *m* Toeplitz matrix over 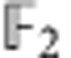. There are two failure events associated with *G*: the minimum distance *δ* being not as large as promised (which results in less-than-expected worst-case performance) and the matrix *G* being not full rank (which can cause two different inputs to be mapped to the same output, leading to a minimum distance of *δ*=0). We will show that, for any fixed rate *R* <1−*H*_2_(*δ*), the probabilities of both failure events decreases exponentially with the output size *m* and can thus be neglected for sufficiently large *m*.

*Theorem 1* ref. 50. Let 
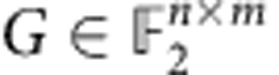
 be a Toeplitz matrix chosen uniformly at random. Let *δ*_min_(*G*) be the minimum distance of the linear code with *G* as generator matrix. Then, for any *δ*∈(0,1/2),


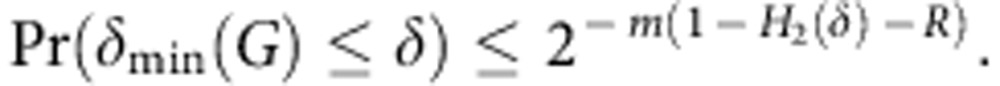


In particular, if *R*=1−*H*_2_(*δ*)−*ε*, for some *ε*>0, then


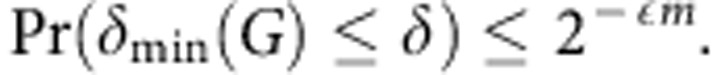


The above theorem guarantees that, if we sacrifice an arbitrarily small quantity *ε* of the rate with respect to the Gilbert–Varshamov bound (that is, we set *R*=1−*H*_2_(*δ*)−*ε*), the probability of obtaining an incorrect minimum distance decreases exponentially with the output size. For example, for a value of *m*=10^7^ and *ε*=10^−3^, this probability is <10^−104^.

*Theorem 2*. Let 
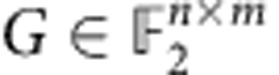
 be a Toeplitz matrix chosen uniformly at random. Then,


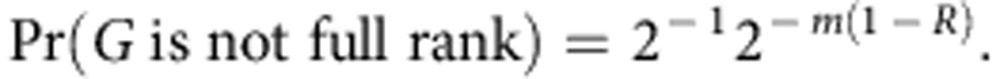


Theorem 2 is an immediate consequence of Theorem 1 in ref. 51. Once again, this probability decreases exponentially with the output size *m*.

### Detailed experimental results

In Table 3, we report the complete results of our experiment. The dominating source of uncertainty is the uncertainty in the total mean photon number of the signals. This uncertainty is due to the summation of the fluctuations of several devices, such as laser power, VOA and varying loss in the channel. For each input size *n*, we perform a calibration process to determine *μ*. In this process, with a proper value of VOA selected from our numerical optimization, the referee sends out around 10^7^∼10^8^ pulses to Alice and Bob. From the total detection counts on *D*_0_ and *D*_1_ and the pre-calibrated losses (Table 1), we estimate the *μ*. We repeat this calibration process a few rounds and obtain the mean value and the s.d. for *μ*. These results are shown in the second column of Table 3. For all tested cases, the uncertainty in mean photon number was below 4%.

Note that the mean photon numbers for Alice and Bob are unequal. This is because in the implementation, to guarantee a good interference visibility, we carefully control the attenuations such that the light from Alice and the light from Bob have the same amplitude when they interfere at the referee. Since the attenuations from Alice to the referee and from Bob to the referee are unequal (Table 1), we choose unequal mean photon numbers for Alice and Bob.

From our model of the protocol, we use the uncertainty in the mean photon number to directly calculate an uncertainty for the quantum transmitted information as well as for the error probability of the protocol. As it can be seen from Table 3, all error probabilities are compatible with the system operating below the target value of *ε*=5 × 10^−5^. In addition, we have included the average values observed for the number of clicks in detector *D*_1_ for equal and different inputs, as well as the threshold values used by the referee.

Finally, we estimate the effect of detector dead times in our experiment as follows. For each input size, we can calculate the probability *p* that an individual pulse leads to a click in detector *D*_1_. In our set-up, after a click occurs, the following 50 pulses are blocked by the detector and cannot be registered. The probability *p*′ that a click occurs for these 50 pulses is given by *p*′=1−(1−*p*)^50^≈50*p*. This number is very small whenever *p* is small, as is the case in our experiment. For instance, for an input size of *n*=1.42 × 10^8^, the expected number of blocked clicks is ∼0.1% of the total expected clicks. Therefore, this effect is negligible compared with fluctuations in the mean photon number, which is of the order of 4%.

## Additional information

**How to cite this article:** Xu, F. *et al.* Experimental quantum fingerprinting with weak coherent pulses. *Nat. Commun.* 6:8735 doi: 10.1038/ncomms9735 (2015).

## Figures and Tables

**Table 1:** Parameters measured in the implementations.

***η***_**AR**_	***η***_**BR**_	***η***_**det**_	***p***_**dark**_	***ν***
3 dB (2.36 dB)	1.5 dB (1 dB)	20.0%	(3.5±0.2) × 10^−6^	(99±0.5)%

The overall loss between the output of Alice's VOA and the input to the referee's detectors is given by the parameter *η*_AR_. Similarly, *η*_BR_ defines the overall loss between the output of Bob's phase modulator and the referee's detectors. Both *η*_AR_ and *η*_BR_ are carefully characterized in ID-500 (Clavis2). The other parameters are the detector's quantum efficiency *η*_det_, dark count rate per pulse *p*_dark_ for each detector, and system visibility *ν*, which are nearly the same for ID-500 and Clavis2.

**Table 2:** The performance of the encoder for different input sizes, using a computer with a quad-core i7-4770 *@*3.4 GHz CPU and 16 GB RAM.

***n*** **(bit)**	***m*** **(bit)**	**Time (s)**	**Memory (Mbit)**
10^6^	5 × 10^6^	6	52
10^7^	5 × 10^7^	106	733
3 × 10^7^	1.5 × 10^8^	181	1,654
3 × 10^8^	1.5 × 10^9^	4,831	10,000

Running times are acceptable for experimental applications for input sizes as large as *n*=3 × 10^8^.

**Table 3:** Detailed experimental results.

**System**	**Clavis2**	**Clavis2**	**Clavis2**	**ID-500**
*n*	1.53 × 10^6^	1.20 × 10^7^	2.27 × 10^7^	1.42 × 10^8^
*μ*_A_	1,914±68	3,295±118	3,670±131	7,120±254
*μ*_B_	1,398±50	2,407±86	2,681±96	5,014±179
*D*_1,E_	22	277	830	1,939
*D*_1,D_	131	318	954	2,224
*D*_1,th_	49	302	902	2110
*Q*	47,689±1,703	93,152±3,326	108,129±3,860	229,713±8,201
*γ*	0.83±0.02	1.19±0.05	1.41±0.05	1.66±0.06
*ε*	(1.6±0.9) × 10^−9^	(2.3±1.4) × 10^−7^	(6.6±3.7) × 10^−6^	(2.9±1.3) × 10^−5^

The parameter *μ*_A_ is the mean photon number for Alice and *μ*_B_ is the mean photon number for Bob. For the clicks in detector *D*_1_ we report the observed averages for the case of equal inputs *D*_1,E_, different inputs *D*_1,D_ and the threshold value used by the referee *D*_1,th_. As before, *Q* is the upper bound on the quantum transmitted information, *γ* is the quantum advantage and *ε* the error probability of the protocol.
